# Structural polymorphisms and distinct genomic composition suggest recurrent origin and ongoing evolution of B chromosomes in the *Prospero autumnale* complex (Hyacinthaceae)

**DOI:** 10.1111/nph.13778

**Published:** 2015-12-08

**Authors:** Tae‐Soo Jang, John S. Parker, Hanna Weiss‐Schneeweiss

**Affiliations:** ^1^ Department of Botany and Biodiversity ResearchUniversity of ViennaRennweg 14A‐1030ViennaAustria; ^2^ Cambridge University Botanic GardenCambridgeCB2 1JFUK

**Keywords:** B‐chromosome evolution, B‐chromosome painting, fluorescence *in situ* hybridization (FISH), genomic *in situ* hybridization (GISH), polyploids, *Prospero autumnale* complex, rDNA (5S and 35S rDNA), satellite DNA *PaB6*

## Abstract

- Supernumerary B chromosomes (Bs) are genomic parasitic components, originating from the A complement via chromosomal rearrangements, which follow their own evolutionary trajectories. They often contain repetitive DNAs, some shared with regular chromosomes and some newly evolved. Genomic composition, origin and evolution of Bs have been analysed in the chromosomally variable *Prospero autumnale* complex.
- Two rDNAs and a satellite DNA (*PaB6*) from regular chromosomes were mapped to Bs of 26 plants from three diploid cytotypes, their hybrids and polyploid derivatives. In homoploid diploid hybrids, genomic *in situ* hybridization (GISH) allowed B painting with the parental DNAs.
- Bs were structurally variable and highly enriched in 5S rDNA and satDNA *PaB6*, and rarely in 35S rDNA. Eleven combinations of rDNA and *PaB6* localization were observed. The quantities of *PaB6* in Bs and regular chromosomes were not correlated, suggesting amplification mechanisms other than recombination. *PaB6* and 5S rDNA amounts increased with increasing ploidy level. GISH revealed two independent origins of Bs.
- The structural variation, repeat content, repeat‐type fluctuations and differing genomic affinities of Bs in different cytotypes suggest that they represent young proto‐B chromosomes. Bs in *P. autumnale* probably form recurrently as by‐products of the extensive genome restructuring within this chromosomally variable species complex.

## Introduction

Karyotypes of many species contain supernumerary genetic material, either as free B chromosomes (Bs) or inserted on standard chromosomes as supernumerary chromosomal segments (SCSs). SCSs are more frequent in insects than in plants, but are most notable in the monocotyledonous family Hyacinthaceae (Greilhuber & Speta, 1978; Ruiz Rejón & Oliver, 1981; Jamilena *et al*., 1995; Ebert *et al*., 1996; Garrido‐Ramos *et al*., 1998; Weiss‐Schneeweiss *et al*., 2004). B chromosomes, by contrast, have been reported in numerous species of animals, fungi and flowering plants (Jones, 1995; Camacho *et al*., 2000). In plants, they are more common in monocots than in dicots, with hot spots in Liliales and Commelinales (Levin *et al*., 2005). B frequencies in diploids and polyploids are similar (Jones & Rees, 1982; Trivers *et al*., 2004), but are higher in families with large genome sizes (Trivers *et al*., 2004; Levin *et al*., 2005; Jones *et al*., 2008).

B chromosomes do not recombine with the A complement and so are exempted from strictly Mendelian inheritance and follow their own evolutionary trajectories (Camacho *et al*., 2000; Jones *et al*., 2008; Houben *et al*., 2013). Bs are most frequently found in low numbers (0–5), but as many as 34 have been reported in an individual of *Zea mays* (Jones & Rees, 1982; Jones *et al*., 2008). They are usually smaller than the standard complement and vary in size from dot‐like micro‐Bs (Houben *et al*., 1997, 2013; Jones *et al*., 2008) to chromosomes as large as the smallest chromosomes of the regular set (Jones *et al*., 2008). Their size and structure are often stable within taxa (*Secale cereale*: Marques *et al*., 2012, 2013), but plants carrying more than one structural B type are well known (Guillén & Ruiz Rejón, 1984; Parker *et al*., 1991).

The occurrence of Bs in phylogenetically unrelated groups indicates their independent and multiple origins (Levin *et al*., 2005). Several hypotheses have been proposed to explain the origins of Bs from A chromosomes (Levin *et al*., 2005; Sharbel *et al*., 2005; Jones *et al*., 2008; Martis *et al*., 2012; Houben *et al*., 2013; Weiss‐Schneeweiss & Schneeweiss, 2013), with most favouring their origin as a by‐product of chromosomal rearrangements of the regular (A) set of chromosomes stimulated by hybridization or polyploidization (Jones & Houben, 2003; Houben *et al*., 2013). Recent support for this hypothesis has come from the genera *Plantago* (Dhar *et al*., 2002) and, in particular, *Secale* (Martis *et al*., 2012). Newly arisen chromosomal fragments often accumulate sufficient differences in structure and/or chromatin composition to ensure their meiotic isolation from A chromosomes, and establish meiotic and mitotic drive mechanisms to secure their own transmission to the next host generation (Langdon *et al*., 2000; Marschner *et al*., 2007; Jones *et al*., 2008; Banaei‐Moghaddam *et al*., 2012; Klemme *et al*., 2013).

During their evolution, Bs capture coding and noncoding DNA sequences from A chromosomes (Małuszyńska & Schweizer, 1989; Dhar *et al*., 2002; Kubaláková *et al*., 2003; Carchilan *et al*., 2009; Banaei‐Moghaddam *et al*., 2012; Marques *et al*., 2012) and from organellar DNAs (Martis *et al*., 2012; Ruban *et al*., 2014), but novel B‐specific repeats also evolve (Langdon *et al*., 2000; Martis *et al*., 2012; Klemme *et al*., 2013). Despite their abundance, the roles of Bs remain enigmatic, although many different effects on the carrier organism have been demonstrated, including influences on A‐chromosome meiotic pairing (Jones *et al*., 2008; Houben *et al*., 2013).

An attractive system in which to establish patterns of B‐chromosome evolution is the genus *Prospero* (Hyacinthaceae). *P. autumnale*, one of three species of this genus, is itself a complex which includes four evolutionarily well‐established diploid cytotypes (AA, B^5^B^5^, B^6^B^6^, B^7^B^7^; Jang *et al*., 2013). Each cytotype is characterized by a unique combination of basic chromosome number, genome size and pattern of rDNA and satellite DNA *PaB6* distribution (Jang *et al*., 2013; Emadzade *et al*., 2014). Polyploidy is frequent in the complex, resulting in autopolyploids of genome B^7^ (*x *=* *7), most commonly 4*x* and 6*x*, but up to 20*x* (Ainsworth, 1981; Ebert, 1993; Speta, 1993, 2000), and two classes of allopolyploids – of A (*x *=* *7) and B^7^ origin, and of B^6^ (*x *=* *6) and B^7^ origin (Vaughan *et al*., 1997; Jang, 2013). Bs have been reported in three of the four diploid cytotypes (the exception is the most recently evolved cytotype, B^5^B^5^, *x *=* *5; Ruiz Rejón *et al*., 1980; Ebert *et al*., 1996; Taylor, 1997), and in a range of polyploids (Ebert, 1993; Taylor, 1997). The Bs vary in size and structure between and within cytotypes, and between and within individuals (Ruiz Rejón *et al*., 1980; Parker *et al*., 1991; Ebert, 1993; Taylor, 1997).

Recently, molecular tools for analysing the evolution of chromosomes in *Prospero* have been developed (Jang *et al*., 2013; Emadzade *et al*., 2014; Jang & Weiss‐Schneeweiss, 2015), which have allowed construction of a phylogenetic framework of the *P. autumnale* complex. In this study, B‐chromosome structure and repeat composition have been analysed in 26 B‐carrying plants of diploid and polyploid cytotypes in the complex using 35S and 5S rDNA probes, along with a species‐specific and evolutionarily dynamic tandem repeat *PaB6* (Emadzade *et al*., 2014). The degree of amplification of *PaB6* and the rDNAs has been compared between the A complement and the accompanying Bs. A recurrent origin of Bs has been established using genomic *in situ* hybridization (GISH) in B‐carrying diploid hybrids. The mode of B meiotic pairing has also been analysed. The results are discussed in the context of *de novo* origin of Bs in different cytotypes, and in relation to the high amounts of chromosomal restructuring of the regular chromosome sets of *P*. *autumnale*.

## Materials and Methods

### Plant materials

In total, 26 plants of the *P. autumnale* (L.) Speta complex containing Bs were analysed (Table 1). Fifteen were diploid (three of cytotype AA, eight B^7^B^7^, one B^6^B^6^, one hybrid AB^7^, two hybrids B^6^B^7^) and 11 were polyploid (three allopolyploids of B^6^ and B^7^ origin, and eight autopolyploids of genome B^7^). For cytological investigations, root meristems were pretreated with a solution of 0.05% colchicine for 4.5 h at room temperature, fixed in ethanol : acetic acid (3 : 1) for at least 3 h at room temperature and stored at −20°C until use. Young flower buds emerging from the bulb were fixed in ethanol : chloroform : acetic acid (6 : 3 : 1) and stored at −20°C.

**Table 1:** Plant material of *Prospero autumnale* complex studied with detailed voucher information

Cytotype	Locality; collection; accession number	2*n*
Diploids		
AA + 1B	Portugal, Peniche; Parker; H549	15
	Spain, Jaén; Parker; H623	15
AA + 2Bs	Portugal, Peniche; Parker; H560	16
AB^7^ + 3Bs	Spain, Jaén; Parker; H546	17
B^7^B^7 ^+ 1B	Greece, Crete; Speta; H209	15
B^7^B^7 ^+ 2Bs	Montenegro; Speta; H415^a^	16
	Greece, Crete; Speta; H526	16
B^7^B^7 ^+ 4Bs	Greece, Crete; Speta; H537	18
	Greece, Skopelos; Parker; H620	18
B^7^B^7 ^+ 5Bs	Montenegro; Speta; H412^a^	19
B^7^B^7 ^+ 6Bs	Italy, Sicily; Speta; H257	20
	Montenegro; Speta; H413	20
B^6^B^6^ + 1B	Greece, Crete; Speta; H154‐1	13
B^6^B^7^ + 2Bs	Greece, Crete; Weigl; H246	15
	Greece, Crete; Speta; H525	15
Polyploids		
B^6^B^6^B^7^B^7^ + 1B	Greece, Crete; Speta; H213	28
B^6^B^6^B^7^B^7^ + 2Bs	Greece, Crete; Raus; H327	30
B^7^B^7^B^7^B^7^ + 1B	Montenegro; Speta; H384	29
	Spain, Biscay; Parker; H624	29
B^7^B^7^B^7^B^7^B^7^ + 1B	Greece, Crete; Jahn; H339‐1	36
B^7^B^7^B^7^B^7^B^7^ + 3Bs	Greece, Karpathos; Raus; H336	38
B^7^B^7^B^7^B^7^B^7^ + 4Bs	Greece, Crete; Jahn; H159	39
B^7^B^7^B^7^B^7^B^7^B^7^ + 1B	Greece, Crete; Speta; H536	43
B^6^B^6^B^7^B^7^B^7^B^7^ + 3Bs	Greece, Crete; Speta; H121	45
B^7^B^7^B^7^B^7^B^7^B^7^ + 4Bs	Tunisia; Speta; H303	46
	Tunisia; Speta; H405	46

a Material used also for meiotic analyses.

### Karyotyping and fluorescence *in situ* hybridization (FISH)

Chromosome numbers and karyotypes were analysed as described by Jang *et al*. (2013) using standard Feulgen staining. Chromosomal spreads for FISH were prepared by enzymatic digestion and squashing as described in Jang *et al*. (2013). Flower buds of plants with B^7^B^7^ + 5Bs and B^7^B^7^ + 2Bs were digested with 1% cellulase Onozuka (Serva, Heidelberg, Germany), 1% cytohelicase (Sigma‐Aldrich) and 1% pectolyase (Sigma‐Aldrich) for 70 min at 37°C.

Probes used for FISH were as follows: satellite DNA *PaB6* isolated from the B^6^ genome in plasmid pGEM‐T Easy (Emadzade *et al*., 2014); 35S rDNA (18S/25S rDNA) from *Arabidopsis thaliana* in plasmid pSK+; and 5S rDNA from *Melampodium montanum* in plasmid pGEM‐T Easy, directly labelled with biotin or digoxygenin (Roche). The plastid probe represents complete plastid genome of *Vicia faba* (courtesy of Dr J. Macas, CAS, Czech Republic).

Probes were labelled either directly by PCR (5S rDNA and satellite DNA *PaB6*) or using a nick translation kit (35S rDNA and plastid probe; Roche). FISH was performed as described in Jang *et al*. (2013). Digoxygenin was detected with antidigoxygenin conjugated with fluorescein isothiocyanate (5 μg ml^−1^: Roche) and biotin with ExtrAvidin conjugated with Cy3 (2 μg ml^−1^: Sigma‐Aldrich). Preparations were analysed with an AxioImager M2 epifluorescent microscope (Carl Zeiss), images captured with a charge‐coupled device (CCD) camera and processed using AxioVision ver. 4.8 (Carl Zeiss) with only those functions that apply to all pixels of the image equally.

### Genomic *in situ* hybridization

Genomic *in situ* hybridization has been performed in two hybrid individuals, B^6^B^7^ (H246) and AB^7^ (H546), using parental diploid genomes DNA as probes (Table 1). Total genomic DNA from diploid cytotypes AA, B^6^B^6^ and B^7^B^7^ was isolated using the CTAB method (Jang *et al*., 2013) and sheared at 98°C for 5 min. Approx. 1 μg of genomic DNA of each cytotype was labelled using either digoxigenin or biotin nick translation kit (Roche).

Genomic *in situ* hybridization was carried out following the method described by Jang & Weiss‐Schneeweiss (2015) after standard chromosome preparations pretreatment (Jang *et al*., 2013). The hybridization mix for hybrids containing the B^6^ genome was modified by addition of unlabelled satellite DNA *PaB6* monomers. The satellite DNA *PaB6* repeats are present in high copy numbers in cytotype B^6^B^6^ and thus, to block these loci and increase GISH performance (noise‐to‐signal ratio), the excess of unlabelled *PaB6* monomers was added to the GISH hybridization mix (Emadzade *et al*., 2014; Jang & Weiss‐Schneeweiss, 2015). Briefly, the hybridization mix included 10% dextran sulphate (Sigma‐Aldrich), 0.02 × saline sodium citrate (SSC) buffer, 1% salmon sperm (Sigma‐Aldrich), 20× access of satellite DNA *PaB6* (for hybrid B^6^B^7^containing B^6^ genome) and 3–4 ng μl^−1^ of each genomic DNA probe; 10 μl of hybridization mix was applied per slide. After hybridization, slides were washed three times in 2 × SSC at 42°C for 3 min each. Probes were detected as described for FISH. All preparations were analysed with an AxioImager M2 epifluorescent microscope (Carl Zeiss), and images were captured with a CCD camera and processed using AxioVision v.4.8 (Carl Zeiss) with only those functions that apply to all pixels of the image equally.

## Results

The number of Bs varied from one to six per individual, but most frequently a single B chromosome was present. B morphology was variable with acro‐, submeta‐ and metacentrics (Supporting Information Fig. S1). B length also varied, from 1.75 to 4.79 μm (Table 2). B morphology was rather uniform in AA diploids, but varied significantly between plants possessing the B^7^ genome (Table 2). The most variable Bs were observed in B^7^ autopolyploids (Table 2).

**Table 2:** Basic morphology and length of B chromosomes (Bs) in *Prospero autumnale* complex

Cytotypes	2*n*	Absolute length of B chromosomes (μm)^a^						Type of Bs	Figures	Accession number
		1	2	3	4	5	6			
*P*. *autumnale* complex										
Diploids										
AA + 1B	14 + 1B	2.78^m^						1‐1	1a, 2a	H549
AA + 1B	14 + 1B	2.92^s^						1‐1	–	H623
AA + 2Bs	14 + 2Bs	2.05^m^	2.14^m^					1‐1	1b, 2b	H560
AB^7^ + 3Bs	14 + 3Bs	2.49^s^	2.74^s^	2.76^s^				1‐2	1c, 2c, 4d	H546
B^6^B^6^ + 1B	12 + 1B	2.92^s^						4	1i, 2j	H154‐1
B^6^B^7^ + 2Bs	13 + 2Bs	2.55^s^	2.61^s^					5, 6	1j, 2k, 4c	H246
B^6^B^7^ + 2Bs	13 + 2Bs	3.13^s^	3.33^s^					7	–	H525
B^7^B^7^ + 1B	14 + 1B	3.37^s^						2	1d, 2d	H209
B^7^B^7^ + 2Bs	14 + 2Bs	2.14^s^	2.20^s^					7	4b	H415
B^7^B^7^ + 2Bs	14 + 2Bs	2.80^m^	2.93^m^					7	1e, 2e	H526
B^7^B^7^ + 4Bs	14 + 4Bs	2.50^s^	3.33^a^	3.33^a^	3.33^a^			7	–	H537
B^7^B^7^ + 4Bs	14 + 4Bs	2.71^a^	2.90^a^	2.94^m^	3.20^s^			7	1f, 2f	H620
B^7^B^7^ + 5Bs	14 + 5Bs	1.81^s^	1.88^a^	1.88^s^	1.92^s^	2.28^s^		3	1g, 2g–h, 4a	H412
B^7^B^7^ + 6Bs	14 + 6Bs	2.29^m^	2.42^m^	2.42^m^	2.50^m^	2.50^m^	2.71^m^	5	–	H257
B^7^B^7^ + 6Bs	14 + 6Bs	2.07^a^	2.11^s^	2.21^m^	2.22^a^	2.32^m^	2.34^s^	7	1h, 2i	H413
Polyploids										
B^6^B^6^B^7^B^7^ + 1B	27 + 1B	2.63^m^						8	1p, 2s	H213
B^6^B^6^B^7^B^7^ + 2Bs	28 + 2Bs	1.82^s^	2.11^s^					7	2t	H327
B^7^B^7^B^7^B^7^ + 1B	28 + 1B	1.88^m^						9	1l, 2m	H384
B^7^B^7^B^7^B^7^ + 1B	28 + 1B	4.79^s^						4	1k, 2l	H624
B^7^B^7^B^7^B^7^B^7^ + 1B	35 + 1B	2.02^m^						10	1m, 2n	H339‐1
B^7^B^7^B^7^B^7^B^7^ + 3Bs	35 + 3Bs	1.75^s^	2.47^m^	2.78^s^				8	2o	H336
B^7^B^7^B^7^B^7^B^7^ + 4Bs	35 + 4Bs	3.00^m^	3.05^s^	3.21^s^	3.27^s^			7	1n	H159
B^7^B^7^B^7^B^7^B^7^B^7^ + 1B	42 + 1B	3.29^m^						11	1o, 2p	H536
B^6^B^6^B^7^B^7^B^7^B^7^ + 3Bs^2^	42 + 3Bs	–	–	–				5	2u	H121
B^7^B^7^B^7^B^7^B^7^B^7^ + 4Bs^2^	42 + 4Bs	–	–	–	–			7	2q	H303
B^7^B^7^B^7^B^7^B^7^B^7^ + 4Bs	42 + 4Bs	2.43^m^	2.54^m^	2.91^m^	3.10^m^			7	2r	H405

a Chromosome types (indicated in superscript): a, acrocentric; m, metacentric; and s, submetacentric; ^2^Bs of these individuals were not measured because it was impossible to identify them among all chromosomes in Feulgen‐stained preparations.

### Tandem repeats in Bs

**Figure 1:**
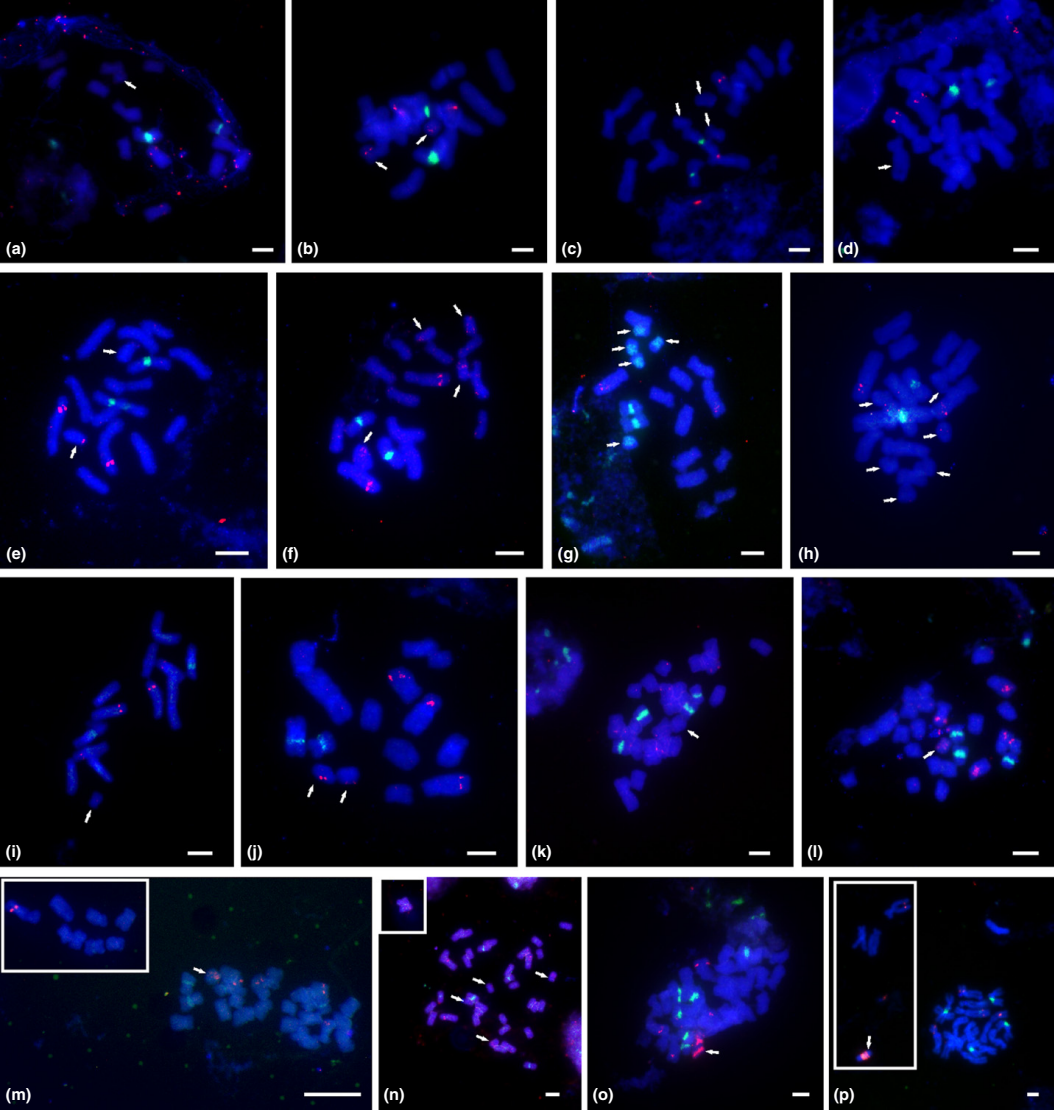
Localization of 35S (green) and 5S rDNA loci (red) in B chromosomes of diploid (a–j) and polyploid (k–p) individuals of the *Prospero autumnale* complex. (a) AA + 1B (H549); (b) AA + 2Bs (H560); (c) AA + 3BS (H546); (d) B^7^B^7^ + 1B (H209); (e) B^7^B^7^ + 2Bs (H526); (f) B^7^B^7^ + 4Bs (H620); (g) B^7^B^7^ + 5Bs (H412); (h) B^7^B^7^ + 6Bs (H413); (i) B^6^B^6^ + 1B (H154‐1); (j) B^6^B^7^ + 2Bs (H246); (k) B^7^B^7^B^7^B^7^ + 1B (H624); (l) B^7^B^7^B^7^B^7^ + 1B (H384); (m) B^7^B^7^B^7^B^7^B^7^ + 1B (H339‐1); (n) B^7^B^7^B^7^B^7^B^7^ + 4Bs (H159); (o) B^7^B^7^B^7^B^7^B^7^B^7^ + 1B (H536); (p) B^6^B^6^B^7^B^7^ + 1B (H213). Insets in (m), (n) and (p) show chromosomes of the same cell that could not be photographed together using a high magnification objective because they were lying at some distance from the main group of chromosomes. Arrows indicate Bs. Bars, 5 μm.

**Figure 2:**
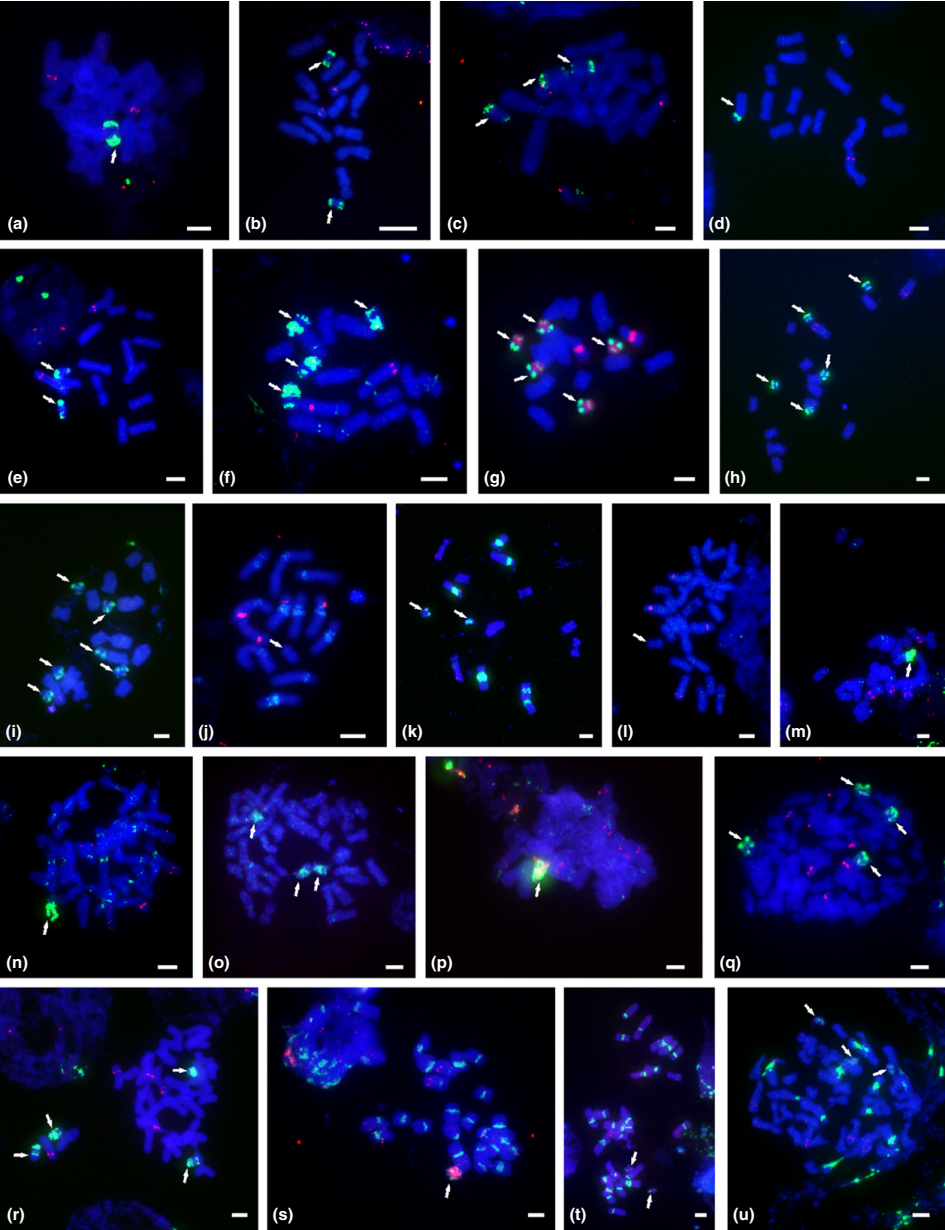
Localization of 5S rDNA (red signals) and satellite DNA *PaB6* loci (green signals) in B chromosomes of diploid (a–k) and polyploid (l–u) individuals in the *Prospero autumnale* complex. Metaphase chromosomes were subjected to fluorescence *in situ* hybridization with 5S rDNA (red in all except for (g) where red depicts 35S rDNA) and 35S rDNA (green). (a) AA + 1B (H549); (b) AA + 2Bs (H560); (c) AA + 3BS (H546); (d) B^7^B^7^ + 1B (H209); (e) B^7^B^7^ + 2Bs (H526); (f) B^7^B^7^ + 4Bs (H620); (g, h) B^7^B^7^ + 5Bs (H412); (i) B^7^B^7^ + 6Bs (H413); (j) B^6^B^6^ + 1B (H154‐1); (k) B^6^B^7^ + 2Bs (H246); (l) B^7^B^7^B^7^B^7^ + 1B (H624); (m) B^7^B^7^B^7^B^7^ + 1B (H384); (n) B^7^B^7^B^7^B^7^B^7^ + 1B (H339‐1); (o) B^7^B^7^B^7^B^7^B^7^ + 3Bs (H336); (p) B^7^B^7^B^7^B^7^B^7^B^7^ + 1B (H536); (q) B^7^B^7^B^7^B^7^B^7^B^7^ + 4Bs (H303); (r) B^7^B^7^B^7^B^7^B^7^B^7^ + 4Bs (H405); (s) B^6^B^6^B^7^B^7^ + 1B (H213); (t) B^6^B^6^B^7^B^7^ + 2Bs (H327); (u) B^6^B^6^B^7^B^7^B^7^B^7^ + 3Bs (H121). Arrows indicate Bs. Bars, 5 μm.

**Figure 3:**
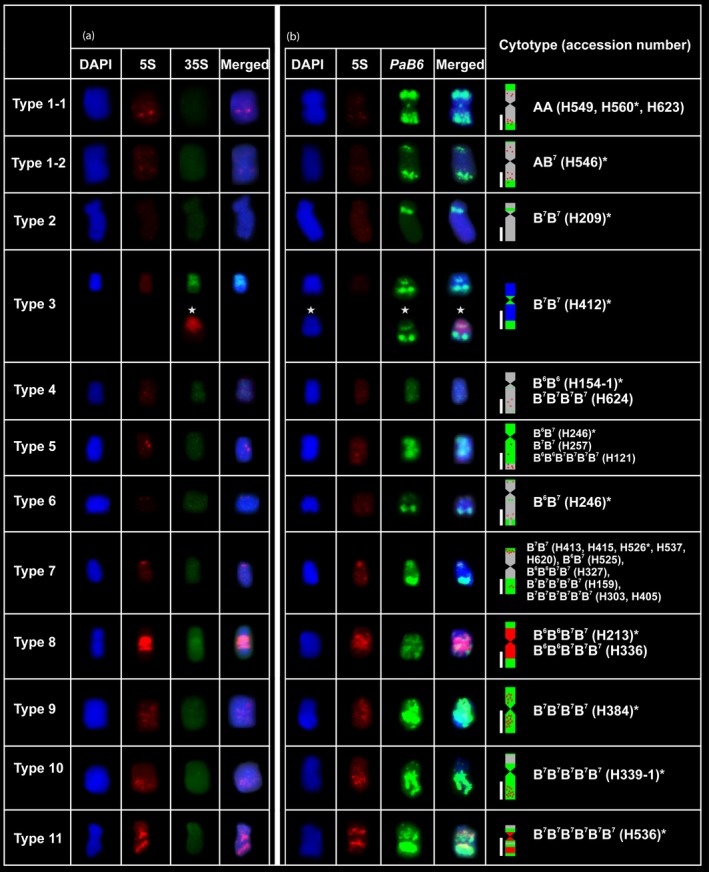
Repetitive DNA distribution in different types of B chromosomes in the *Prospero autumnale* complex. The individual used as the source of chromosomes depicted in the figure is marked with asterisk. (a) Colocalization of 5S (red) and 35S rDNA (green). (b) Colocalization of 5S rDNA (red) and satellite DNA *PaB6* repeats (green). Whole chromosomes were counterstained with 4′,6‐diamidino‐2‐phenylindole (DAPI; blue). Bars, 1 μm.

35S rDNA, 5S rDNA and satellite DNA *PaB6* tandem repeats were mapped in mitotic and meiotic chromosomes of the standard complements and in their Bs (Tables 1, 2; Figs 1, 2, 3). Hybridization using plastid DNA regions (complete plastid DNA from *Vicia faba*) produced no discernible signals (Fig. S2).

#### 35S and 5S rDNA repeats

Only one plant (H412; cytotype B^7^B^7^) carried Bs with detectable 35S rDNA repeats. All five acrocentric Bs in this plant possessed signals spread over their whole short arms and part of their long arms (Figs 1g, 3 type 3). No secondary constrictions were visible.

5S rDNA, by contrast, was detected in almost all the remaining Bs, with the exception of Bs in two B^7^B^7^ diploids (Fig. 3). The signals were weak and dot‐like in diploids (Figs 1a–j, 3 types 1–7) with the copy number increasing with ploidy level (Figs 1o,p, 3 types 8, 11). Only one B type had a clearly defined and very large 5S rDNA locus. It occupied an extensive pericentric region, which constituted more than half the B (found in allotetraploid and allopentaploids of B^6^ and B^7^ genomes; Figs 1p, 3 type 8). 5S rDNA was not detected in the Bs carrying 35S rDNA (Fig. 3 type 3).

#### Satellite DNA *PaB6*

Satellite DNA *PaB6* was found in Bs of diploids and polyploids. Bs in polyploid backgrounds carried higher copy numbers of satDNA and the distribution was more variable (Table 2; Fig. 2). These differences were particularly evident in the B^7^ genome, in which entire Bs were painted by *PaB6* in autopolyploids (Figs 2, 3). *PaB6* and 5S rDNA were often amplified together in Bs, but usually occupied separate chromosomal regions (Fig. 3). The five Bs carrying 35S rDNA (in H412 of cytotype B^7^B^7^) also carried *PaB6* in a distinct, nonoverlapping, chromosomal domain (Fig. 3 type 3).

In Bs of diploids, copy number and distribution of *PaB6* were usually variable. Bs in AA diploids and in the AB^7^ hybrid were uniform in structure and in tandem repeat distribution. They were enriched in *PaB6* (Fig. 3) in subterminal regions of both chromosomal arms (Figs 2a–c, 3 type 1a,b) although their amounts differed (Fig. 3). The B in the B^6^B^6^ diploid, by contrast, possessed a very low copy number of *PaB6* (Figs 2j, 3 type 4), despite the high amount of this repeat in the regular complement (Figs 2j, 3 type 4).

B chromosomes in the B^7^ diploids were variable (Figs 2d–i, 3 types 2, 3, 5, 7), with the most common type mildly enhanced for *PaB6* in the pericentric region but highly enriched subterminally in the long arm (types 3, 7). Bs of one B^7^B^7^ plant exhibited very high levels of *PaB6* amplification, with nearly the whole B covered (type 5). In plant H209 of B^7^B^7^, a putative, large B showed clear pericentric localization of *PaB6* typical of chromosomes of the standard complement, but with a much higher copy number (Figs 2d, 3 type 2); it lacked interstitial and distal *PaB6* loci. One B^6^B^7^ hybrid carried two different B‐variants (H246, Figs 2k, 3, types 5, 6), while the other carried the B type typical of B^7^B^7^ diploids (type 7).

Overall, Bs in polyploids possessed the highest amounts of *PaB6*, which typically painted whole Bs (types 9–11), although with signals slightly less prominent in short arms. One B^7^ autotetraploid (H624) carried a B with a very low level of *PaB6* amplification, distributed in small foci over the whole chromosome (Fig. 3 type 4). B^6^B^7^ allopolyploids (4*x* and 6*x*) mainly carried the Bs characteristic of B^7^ diploids (types 5 and 7). One new variant (type 8), however, was found, in which *PaB6* was amplified in multiple small loci over the whole chromosome length (H213, 4*x*; H336, 5*x*).

### B chromosomes in diploids and polyploids

Based on the patterns given by the three DNA repeats, 11 distinct B types have been identified in only 26 plants of the *P. autumnale* complex (Fig. 3). Seven B types have been identified in 14 diploids (types 1–7) and three of these (types 4, 5, 7) were also found in polyploids, with type 7 being the most prevalent (four occurrences). Four further B types were found uniquely in the sample of only five polyploids (types 8–11). A major difference between Bs in diploids and polyploids was the enhanced amplification of the 5S rDNA and satellite DNA repeats in polyploids.

### Meiotic behaviour of Bs in diploids

**Figure 4:**
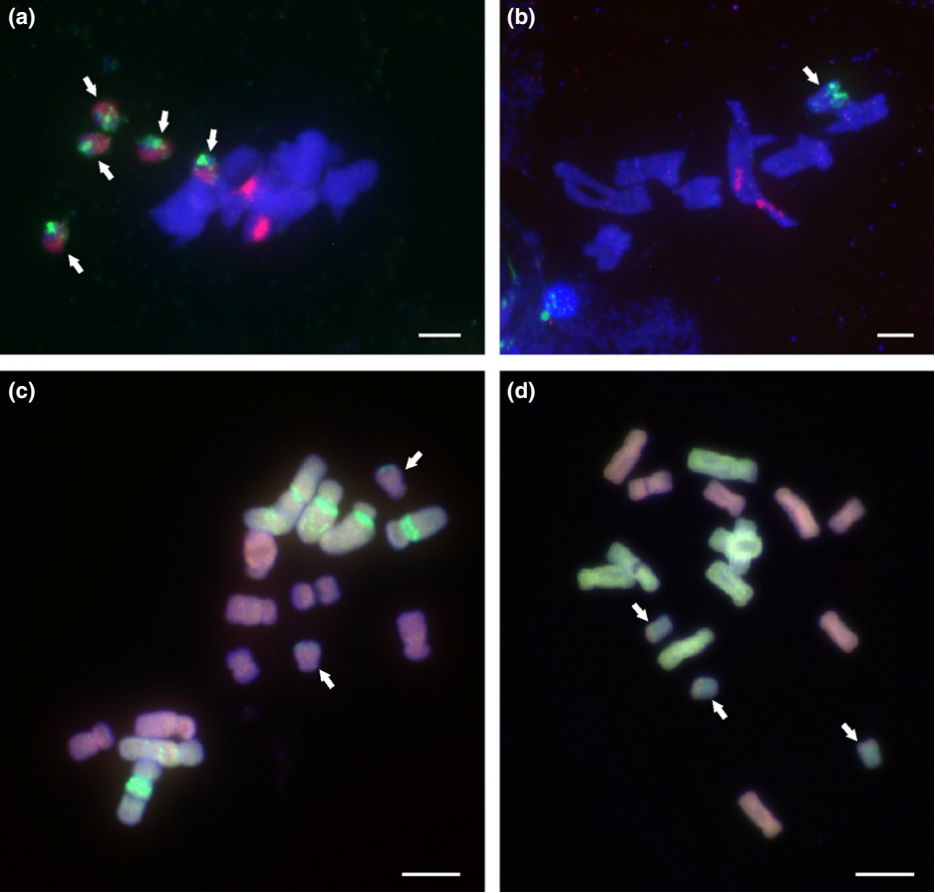
Fluorescence *in situ* hybridization (FISH) with rDNA (red) and satellite DNA *PaB6* (green), and genomic *in situ* hybridization (GISH) in the *Prospero autumnale* complex. (a, b) Meiotic metaphase I of two *Prospero autumnale* individuals carrying B chromosomes: (a) cytotype B^7^B^7^ with five Bs (H412; 35S rDNA in red; *PaB6* in green); (b) cytotype B^7^B^7^ with two Bs (H415; 5S rDNA in red; *PaB6* in green). (c, d) GISH in diploid homoploid hybrid individuals carrying Bs: (c) localization of B^6^ (green) and B^7^ (red) genomic DNA in B^6^B^7^ with two Bs (H246); (d) localization of A (green) and B^7^ (red) genomic DNA in AB ^7^ with three Bs (H546). Arrows indicate Bs. Bars, 5 μm.

Meiotic behaviour of Bs in two B^7^ diploids revealed different patterns of pairing (Fig. 4). In neither did the Bs pair with standard chromosomes. The five Bs in H412, enriched in *PaB6* and 35S rDNA, were present as univalents (22 cells; Fig. 4a). By contrast, the two Bs of H415, with *PaB6* and 5S rDNA repeats, regularly formed a bivalent (26 cells; Fig. 4b).

### Genomic DNA affinities of Bs

The relationships of Bs to parental genomes were examined using GISH in two different diploid hybrids which were first‐generation crosses – B^6^B^7^ with two Bs (H246; types 5, 6) and AB^7^ with three Bs (H546; type 1–2). These Bs, therefore, had not undergone meiosis in their current genomic backgrounds. Hybridizations with labelled parental genomic DNAs were carried out.

The Bs in the B^6^B^7^ hybrid had a higher affinity for the DNA probe of the B^7^ than the B^6^ genome; they also carried *PaB6* in subterminal positions on their long arms (Fig. 4c). The Bs of the AB^7^ hybrid were painted by the A genome alone, although the intensity of hybridization was slightly lower than that displayed by the standard A chromosomes (Fig. 4d).

## Discussion

### Structural variation of Bs in the *P. autumnale* complex

B chromosomes in *P. autumnale* are, with a single exception, smaller than the smallest standard chromosome (Ainsworth, 1981; Ainsworth *et al*., 1983; Table 2). Bs, highly variable in structure and heterochromatin composition, have previously been reported in diploids and polyploids of *P. autumnale* (Ruiz Rejón *et al*., 1980; Parker *et al*., 1991; Ebert, 1993; Taylor, 1997) and the Bs in the current study represent a subset of those described. Telocentrics, acrocentrics and metacentrics have previously been documented (Ruiz Rejón *et al*., 1980; Hong, 1982; Guillén & Ruiz Rejón, 1984; Parker *et al*., 1991; Ebert *et al*., 1996), but no telocentrics were included in the current sample. As many as 11 B types were observed based on B morphology but mainly on the distribution of tandem repeats in the Bs (Fig. 3) of the 26 individuals analysed here, representing all available cytotypes. The previous studies of Bs have described numerical and morphological variation using standard Feulgen staining, but heterochromatin content and distribution were established in only two plants (one B^6^B^6^, one B^7^B^7^; Ebert *et al*., 1996).

The Bs of the AA cytotype studied here are all rather uniform in structure and nearly identical to the common variant previously reported in AA diploids from Spain and Portugal (Parker *et al*., 1991). Cytotype B^7^B^7^ showed the highest amount of structural B variation, both here and in previous investigations (Ainsworth, 1981; Ebert, 1993; Ebert *et al*., 1996; Taylor, 1997). While this diversity might simply reflect the higher number of B^7^B^7^ plants examined, it may also indicate multiple, independent origins of Bs within the B^7^ genome lineage, which is widespread across the whole Mediterranean Basin (Vaughan *et al*., 1997; Jang *et al*., 2013). Structural polymorphisms of Bs have been reported in many other organisms, both plant and insect (Lopez‐Leon *et al*., 1993; Jones, 1995). In *Allium schoenoprasum*, for example, 13 different B forms have been described in Welsh populations (Bougourd & Parker, 1979; Holmes & Bougourd, 1989), while in two species of *Aster*, as many as 29 variants have been identified in studies involving hundreds of populations (reviewed in Jones, 1995).

### B chromosomes and repetitive DNA accumulation

Studies of heterochromatin in Bs of two B^7^B^7^ plants reported submetacentrics with pericentric dot‐like C bands, and acrocentrics with either blocks or dot‐like small heterochromatic loci in subterminal, interstitial and/or pericentromeric regions (Ebert, 1993). This corresponds to part of the B variation seen here. Ebert *et al*. (1996) also reported Bs in B^6^B^6^ plants, structurally similar to those found in the current study. These Bs had no C bands, and so agree with observations made here that Bs in the B^6^ cytotype have low numbers of tandem repeats of rDNA and *PaB6*.

Large insertions of plastid and mitochondrial DNA sequences are sometimes detected in plant Bs (Martis *et al*., 2012; Klemme *et al*., 2013; Ruban *et al*., 2014), although none have been found in *P. autumnale* Bs. Bs frequently share repeat families with the A complement, but also accumulate B‐specific families (Langdon *et al*., 2000; Dhar *et al*., 2002; Marques *et al*., 2012; Martis *et al*., 2012). Bs of *P. autumnale* share three repeat families with the A complement – 5S and 35S rDNAs and the *Prospero*‐specific satellite DNA *PaB6*. Although *PaB6* is evolutionarily very dynamic and has accompanied diversification of diploid cytotypes and their polyploid derivatives in *P. autumnale* (Jang, 2013; Emadzade *et al*., 2014), amplification levels in Bs are not correlated with those in their respective standard complements. No B‐specific repeats have yet been found in *P. autumnale* Bs, although analyses to detect them are under way.

In the phylogenetically well‐defined cytotype AA, all Bs have similar repetitive DNA content and distribution, which parallel their structural uniformity. The A genome is geographically restricted to the Iberian Peninsula and may have become isolated from the B^7^ genome during the most recent glacial era (Parker *et al*., 1991; Jang *et al*., 2013). This may have led to the fixation of a single type of B (Parker *et al*., 1991). By contrast, eight different B‐types were found in the widespread B^7^B^7^ diploid and its derivative polyploids. The five B types found exclusively in B^7^ autopolyploids might have originated from the most common B type found in diploids, by large‐scale amplification of *PaB6* and/or 5S rDNA and subsequent divergence.

B chromosomes of polyploids generally had higher *PaB6* and 5S rDNA amplification levels than those of diploids, suggesting a positive correlation between these two traits. This may be related to the higher amounts of genome restructuring in polyploids than in diploids within this species complex (Jang, 2013; Weiss‐Schneeweiss *et al*., 2013). No comparable data yet exist for other plant genera.

The lack of meiotic pairing between Bs and standard chromosomes precludes recombination as a direct mechanism mediating spread of *PaB6* and rDNA repeats in Bs. Extrachromosomal circular DNAs may facilitate spread and homogenization of tandem repeats (eccDNAs; Cohen *et al*., 2008; Navrátilová *et al*., 2008), and these should be sought within *Prospero*. The widespread presence of 5S rDNA repeats in Bs suggests that they originated from standard chromosomes carrying 5S rDNA (chromosome 1 and/or 2; Jang *et al*., 2013). However, extrachromosomal circular DNAs have again been suggested as a mechanism of spread of this repeat (Cohen *et al*., 2008, 2010).

### On the origin of Bs in *P. autumnale*

The *P. autumnale* complex is chromosomally very variable and dynamic so high degrees of variation in structure and repeat composition of Bs are immediately suggestive of their independent and multiple origins. However, structural and repeat content polymorphisms have also been demonstrated in plant groups in which the Bs were shown to be of a single, unique origin (Martis *et al*., 2012; Marques *et al*., 2013; Ruban *et al*., 2014).

The *P. autumnale* cytotypes are genomically very similar to each other and their repetitive DNA fractions share > 90% similarity (Emadzade *et al*., 2014; H. Weiss‐Schneeweiss *et al*., unpublished). The standard GISH technique was ineffective in resolving such similar parental genomes, but recently a modified formamide‐free GISH technique has allowed clear identification of parental genomes in hybrids (Jang & Weiss‐Schneeweiss, 2015). This formamide‐free GISH was used in this study to test for the recurrent origin of Bs using two diploid homoploid hybrids – B^6^B^7^ (with type 5 Bs) and AB^7^ (with type 1–2 Bs). The results indicated different genomic affinities of these two B types, one similar to the B^7^ genome (type 5; B^6^B^7^ hybrid) and the other to the A genome (type 1–2; AB^7^ hybrid). It has so far proved impossible to use GISH on Bs in polyploids, because of the high levels of amplification of *PaB6* and 5S rDNA. Both repeats produce strong signals which obscure the genomic affinities of Bs even if they are used in high concentrations as unlabelled blocking DNA (Jang & Weiss‐Schneeweiss, 2015).

The GISH results give a conservative estimate of two origins of Bs in diploids, one in AA cytotype (type 1) and one in B^7^ cytotype (type 5); other types could potentially have evolved from type 4 by differential repeat amplification or removal. However, an independent origin is also likely for the B in the B^6^ cytotype (type 4), perhaps indicated by the lack of repeats in this B. This study thus provides the first evidence of recurrent B formation in *P. autumnale* and investigation will now be extended to the population level.

The genus *Prospero* has been estimated to originate 6–7 million yr ago (Ma) (Ali *et al*., 2011), and *P. autumnale* only *c*. 1 Ma (K. Emadzade *et al*., unpublished). Neither Bs nor polyploidy have been found in the two other *Prospero* species, *Prospero obtusifolium* and *Prospero hanburyi* (Jang *et al*., 2013). Thus, the Bs in *Prospero* are probably evolutionarily very young. Despite this, the Bs are well established and widespread, and do not pair at meiosis with the regular chromosome complement.

B chromosomes in polyploids of *P. autumnale* were mainly found in the widespread and common autopolyploids of genome B^7^, or else in allopolyploids involving B^7^. Whether these Bs share a common origin or have originated independently and accumulated the same repeats during polyploid genome restructuring will require further study, perhaps using the new formamide‐free GISH technique.

*Prospero autumnale* is a young and chromosomally extremely variable species complex, with diploid cytotypes evolving from an ancestral genome on different evolutionary timescales. It has undergone, and continues to undergo, chromosomal fusions, inversions and translocations accompanied by changes in the repeatome and DNA amounts (Ainsworth *et al*., 1983; Jang *et al*., 2013). Bs perhaps represent recent and recurrent by‐products of these extensive chromosomal changes, thus following the mode of B‐chromosome formation elegantly demonstrated in *Secale* (Martis *et al*., 2012) or cichlid fish (Valente *et al*., 2014). Polyploidy provides an additional level of chromosomal complexity in *P*. *autumnale*, which may itself provide a stimulus to genome restructuring and B generation.

### Conclusions

B chromosomes in the chromosomally variable species complex *P. autumnale* provide an excellent system in which to address their origin and evolution. The extent of variation of Bs is extraordinarily high and only the minimal level of variation has so far been assessed. The data suggest a recurrent origin of proto‐B chromosomes in *P. autumnale*, which are then dynamically evolving. This hypothesis will now be tested on a larger scale, involving populational and biogeographical analyses. Analyses of meiotic and postmeiotic behaviour could offer insights into the modes and mechanisms of B transmission.

## Author contributions

H.W‐S. and T‐S.J. planned and designed the research. T‐S.J. performed experiments. T‐S.J., J.S.P. and H.W‐S. analysed the data and wrote the manuscript.

## Supporting information

Please note: Wiley Blackwell are not responsible for the content or functionality of any supporting information supplied by the authors. Any queries (other than missing material) should be directed to the *New Phytologist* Central Office.

**Fig. S1** Structure of B chromosomes in 24 of 26 analysed individuals of *Prospero autumnale*.
**Fig. S2** Localization of plastid DNA sequences and satellite DNA *PaB6* loci in B chromosomes of the *Prospero autumnale* complex.
