# Supplementary material for: Structural polymorphisms and distinct genomic composition suggest recurrent origin and ongoing evolution of B chromosomes in the Prospero autumnale complex (Hyacinthaceae)
Source: New Phytol. 2015 Dec 8;210(2):669–79. doi: 10.1111/nph.13778 (PMC4949986; doi:10.1111/nph.13778)

New Phytologist

***New Phytologist* Supporting Information**

Article title: **Structural polymorphisms and distinct genomic composition suggest recurrent origin and ongoing evolution of B chromosomes in the *Prospero autumnale* complex (Hyacinthaceae**)

Authors: Tae-Soo Jang, John S. Parker and Hanna Weiss-Schneeweiss

Article acceptance date: 28 October 2015

The following Supporting Information is available for this article:
Fig. S1 Structure of B-chromosomes in 24 of 26 analysed individuals of *Prospero autumnale*.

Fig. S2 Localization of plastid DNA sequences and satellite DNA PaB6 loci in B-chromosomes of the *Prospero autumnale* complex.

Fig. S1 Structure of B-chromosomes in 24 of 26 analysed individuals of *Prospero autumnale*. Reliable identification of Bs in two remaining polyploid individuals in Feulgen stained preparations was impossible. Bar, 5 µm.

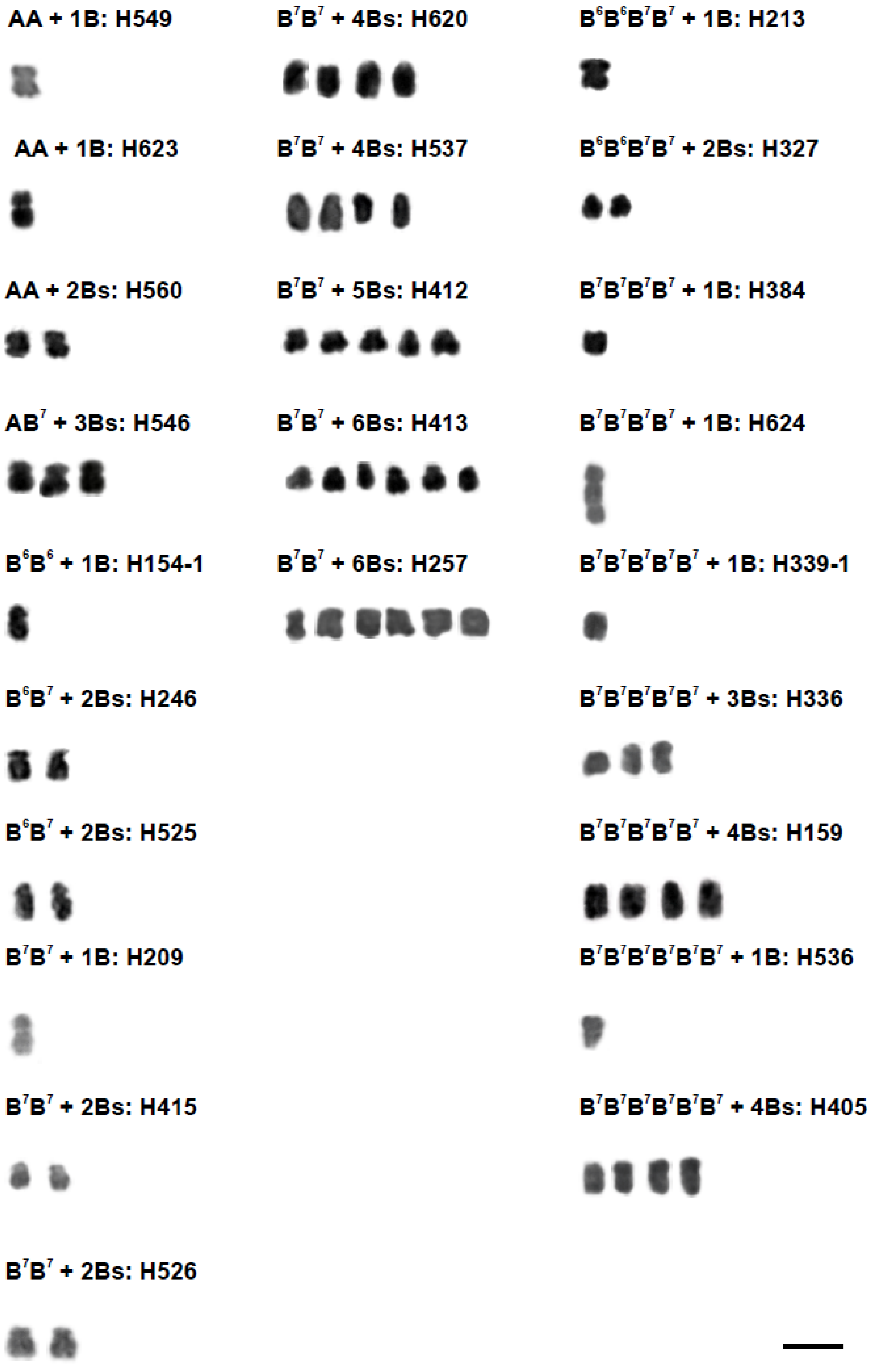

Fig. S2 Localization of plastid DNA sequences and satellite DNA *PaB6* loci in B-chromosomes of the *Prospero autumnale* complex. (a) AA + 1B (H549); (b) AB^7 + 3Bs (H546); (c) B^7B^7 + 2Bs (H526); (d) B^7B^7 + 5Bs (H412). (a1–d1) Merged images of the three channels; (a2–d2) FISH with plastid DNA probe (red); (a3–d3) FISH with satellite DNA *PaB6* probe (green); (a4–d4) DAPI (4',6-diamidino-2-phenylindole) staining. Arrows indicate Bs. Bars, 5 µm.

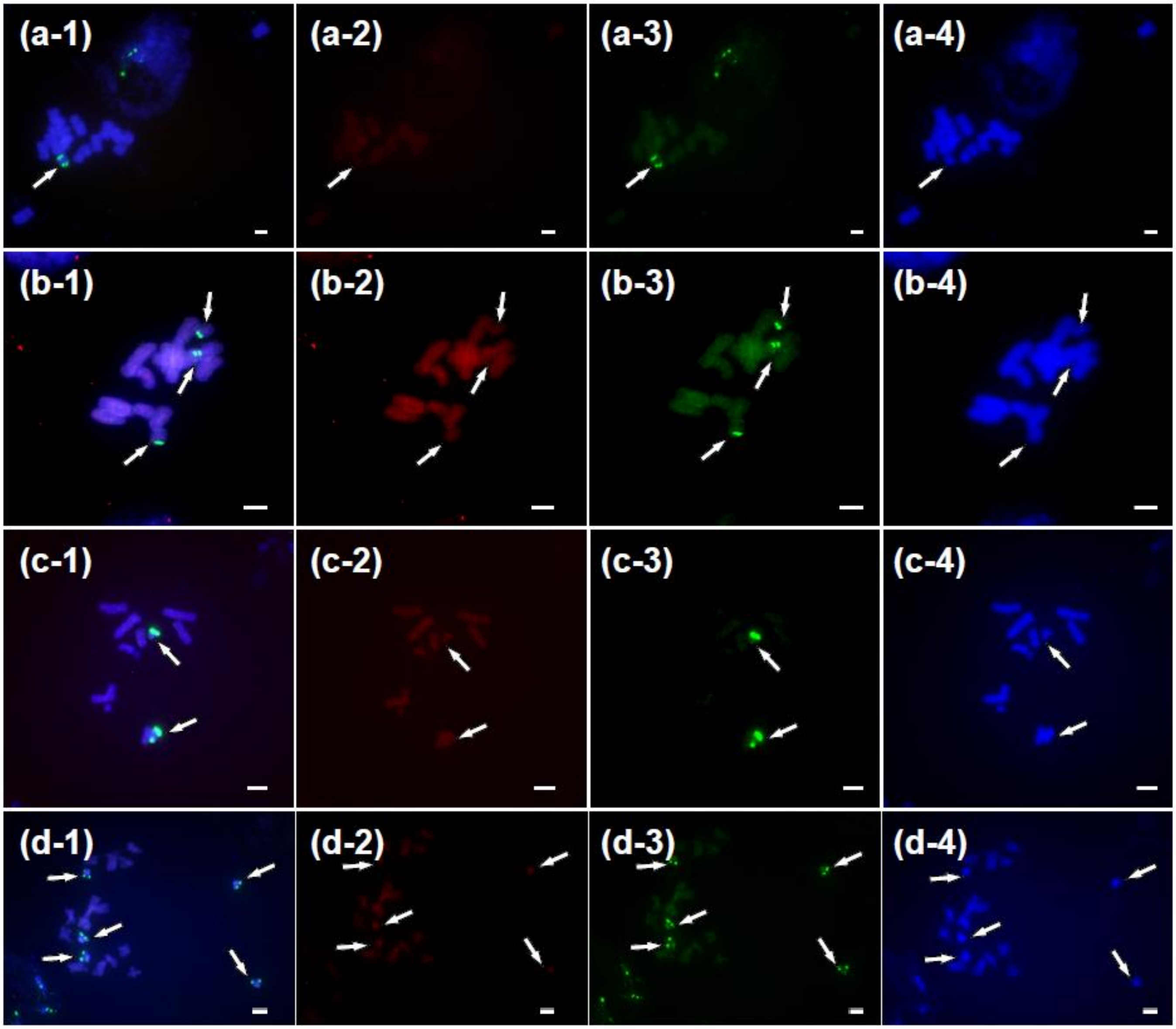

Supplement: Supplementary file 1 — Fig. S1 Structure of B chromosomes in 24 of 26 analysed individuals of Prospero autumnale. Fig. S2 Localization of plastid DNA sequences and satellite DNA PaB6 loci in B chromosomes of the Prospero autumnale complex. [file NPH-210-669-s001.pdf]
